# State-of-the-art of multidisciplinary approach of bone metastasis-directed therapy: review and challenging questions for preparation of a GEMO practice guidelines

**DOI:** 10.1007/s10555-025-10262-6

**Published:** 2025-04-12

**Authors:** Emmanuel Mesny, Nicolas Martz, Nicolas Stacoffe, Frédéric Clarençon, Matthias Louis, Nacer Mansouri, François Sirveaux, Sébastien Thureau, Jean-Christophe Faivre

**Affiliations:** 1 Radiation Oncology Department, Hospices Civils de Lyon, CHLS, Lyon, France; 2 Radiation Oncology Department, Institut de Cancérologie de Lorraine–Alexis-Vautrin, Vandœuvre-Lès-Nancy, France; 3 Radiology Department, Hospices Civils de Lyon, CHLS, Lyon, France; 4 Department of Interventional Neuroradiology, AP-HP La Pitié-Salpêtrière, Paris, France; 5 Radiology Department, CHU de Nancy, Nancy, France; 6 Neurosurgery Department, CHU de Nancy, Nancy, France; 7 Orthopedic Surgery Department, CHU de Nancy, Nancy, France; 8 Radiation Oncology Department and Litis Quantif, EA, 4108 Unity, Centre Henri Becquerel, Rouen, France

**Keywords:** Bone metastases, Radiotherapy, Surgery, Interventional radiology, Skeletal-related event

## Abstract

Bone is a common secondary site of dissemination during the course of cancer. Bone metastases (BM) can be associated with skeletal-related events (SRE) such as disabling pain, hypercalcemia, and bone instability that leads to pathological fractures or spinal cord compression. SRE contribute to high morbidity as well as, mortality, and have a negative economic impact. Modern management of BM integrates focal treatments (such as radiotherapy, surgery, and interventional radiology), orthoses, and antiresorptive and systemic oncological treatment. The choice of a metastasis-directed therapy depends on the objective of the treatment, the patient characteristics, and the complete assessment of the bone lesion (pain, neurological risk, and instability). In the narrative review present herein, we aim to provide an updated summary of the literature, with description of the advantages and disadvantages of current and emerging strategies in the multimodal treatment of BM and, based on these data, an updated algorithm for the management of BM.

## Introduction

Bone is a common secondary site of dissemination during the course of cancer [1]. Bone metastases (BM) can be associated with skeletal-related events (SRE) such as disabling pain, hypercalcemia, and bone instability that leads to pathological fractures or spinal cord compression or other symptoms requiring an urgent intervention such as surgery or radiotherapy [2]. SRE contribute to high morbidity as well as mortality and have a negative economic impact [3]. The goal of managing metastatic bone disease is to prevent SRE and the associated complications in order to maintain the patient’s quality of life through the maintenance of their activities of daily living by pain relief, prevention, and treatment of fractures or paralysis. The modern multimodal management for BM combines metastasis-directed therapies (MDT) such as surgery, radiotherapy (RT) or radiology, associated with systemic treatment, antiresorptive treatment, and supportive care. Each MDT technique has been individually evaluated in terms of benefits and side effects, but there are no published data comparing these therapies. Moreover, the primary endpoints used to describe the efficacy of each technique are often different. A preliminary decision framework has been published in 2017 to help physicians in the treatment of spinal BM [4]. In the review present herein, we aim to provide an updated summary of the literature, with description of the advantages and disadvantages of current and emerging strategies in the multimodal treatment of BM and, based on these data, an updated algorithm for the management of BM.

## Skeletal-related events (SRE) and fracture risk assessment

The growing access to modern diagnostic tools allows early detection of asymptomatic BM that could be successfully managed with local or systemic treatment to avoid the development of SRE. Computed tomography (CT)-scan is the first-line investigation, but magnetic resonance imaging (MRI) should be performed first in cases of suspected neurological complications (metastatic epidural spinal cord compression (MESCC), radicular compression, and cauda equina syndrome), providing better details for soft tissue or epidural invasion. Other imaging modalities (technetium bisphosphonate bone scintigraphy, positron emission tomography (PET) with ^18^fluorodeoxyglucose (FDG) or other radiotracers) also allow assessment of bone involvement [5].

The management strategy for these lesions is essentially based on fracture risk and neurological risk evaluation. The Spinal Instability Neoplastic Score (SINS) is used to determine the instability risk of spinal BM to decide when to refer the patients to a neurosurgeon. It can also be a useful tool to assess the need of a corset. This score, ranging from 0 to 18, classifies the lesion into 3 categories: stable (≤ 6), potentially unstable (7 to 12), and unstable (≥ 13). It considers the location and nature of the lesion, the presence of mechanical or postural pain, the spinal alignment and vertebral body involvement. Its equivalent for long bones, the Mirels’ score ranging from 4 to 12, also classifies the lesion into 3 groups: low risk (≤ 7), moderate (8), and high risk of fracture (≥ 9) [6]. For long bones, evaluating cortical bone invasion in the entire bone is also essential [7]. Unstable lesions or those at high risk of fracture require multidisciplinary management with the aim of achieving stabilization. The Bilsky classification is another score used to precise the extent of the spinal cord infiltration in case of MESCC [8].

## Objectives of MDT

### SRE prevention

Analgesic and stabilizing purposes or spinal cord compression are common indications for treatment of BM to maintain patient’s activities of daily living. In patients with oligometastatic disease, focal treatment of BM, and the benefit in terms of local control (LC) which results from it, could increase survival.

#### Analgesic effect

Bone metastatic pain is most rapidly relieved with surgery and vertebroplasty but this can cause post-procedure pain of varying duration. Although surgery provides immediate analgesic benefits, due to its invasive nature and associated comorbidities, surgery is reserved for cases of mechanical instability or neurological risk, to prevent SRE, alleviate pain, or improve function [9–11].

Cementoplasty for spine metastases has a rapid analgesic effect (within 24 to 48 h), particularly for mechanical pain caused by fractures, improving quality of life and reducing use of analgesics [12]. For extra-spinal metastases, a recent systematic review noted significant pain relief between pre-and post-operative pain scores [13]. Radiofrequency ablation (RFA), often paired with cementoplasty, shows 44% of pain reduction within the first week and a median overall response rate (RR) for pain reduction of 67% and 74% after a median follow-up of 6 months and 12 months, respectively [14, 15]. Cryoablation (CA) also effectively reduces pain; it has been reported to decrease mean pain scores significantly by 62.5% at 24 h, 70% at 3 months, and 80.9% at 6 months [16], and after CA alone a complete pain RR of 32% and a partial pain RR of 36% [17]. In addition, embolization and electrochemotherapy (ECT) are emerging treatments; ECT has been found to lead to notable pain reduction [18–20].

For RT, the overall RR is approximately 70%, with a complete RR of 30% [21]. Stereotactic body radiotherapy (SBRT) is an emerging technique for pain management, allowing for targeted dose escalation while minimizing exposure to adjacent healthy tissues. Randomized controlled trials comparing three-dimensional radiation therapy (3DRT) to SBRT have found significant benefits for complete pain response at 3 and 6 months and overall pain response at 6 months [22–24] (Table 1). A 2023 meta-analysis found excellent outcomes with palliative SBRT for painful spinal metastases: 87% partial and 51% complete pain RR, with a low frequency of serious adverse effects [25]. The European Society for Therapeutic Radiology and Oncology (ESTRO) recommends SBRT for selected patients with uncomplicated painful spine metastases meeting specific criteria: stable metastases (SINS < 12), minimal to no epidural disease (Bilsky score ≤ 1), involvement of up to 3 contiguous vertebral segments, and a life expectancy > 3–6 months [26]. Additionally, the American Society for Therapeutic Radiology and Oncology (ASTRO) suggests SBRT for patients with a Performance status (PS) 0–2, without prior surgery or neurological symptoms [27].

**Table 1:** Randomized trials investigating SBRT in painful bone metastases

Publication	Phase of trial	Number of patients	Study design (arms)	Efficacy		Toxicity
				3 months	6 months	
Sprave et al., 2018 [28]	Phase II	55	Exp.: 24 Gy in 1 Fr (SBRT) Control: 30 Gy in 10 Fr (3DRT)	**CPR**: 43.5% SBRT, 17.4% 3DRT *(p* = *0.0568)* **PPR**: 26.1% SBRT, 30.4% 3DRT	**CPR**: 52.6% SBRT, 10% 3DRT *(p* = *0.0034)* **PPR**: 21.1% SBRT, 25.0% 3DRT	No CTCAE grade 3 or 4 toxicity No RIM
Nguyen et al., 2019 [29]	Phase II	160	Exp.: 12 or 16 Gy in 1 Fr (SBRT) Control: 30 Gy in 10 Fr (3DRT)	Responders (CPR + PPR): 72% SBRT, 49% 3DRT *(p* = *0.03)*	Responders (CPR + PPR): 68% SBRT, 61% 3DRT *(p* = *0.78)*	**No differences in CTCAE grade 3 toxicity**: Nausea: 1.2% SBRT *vs.* 5.0% 3DRT Vomiting: 0% SBRT *vs.* 2.5% 3DRT Fatigue: 9.9% SBRT *vs.* 5.1% 3DRT Fracture: 1.2% SBRT *vs.* 0% 3DRT
Pielkenrood et al., 2020 [30]	Phase II	110	Exp.: 18 Gy in 1 Fr/30 Gy in 3 Fr/35 Gy in 5 Fr (SBRT) Control: 8 Gy in 1 Fr/20 Gy in 5 Fr/30 Gy in 10 Fr (3DRT)	**PPR**: 40% SBRT, 32% 3DRT *(p* = *0.42)*	/	No CTCAE grade 3 or 4 toxicity
Sahgal et al., 2021 [31]	Phase II/III	229	Exp.: 24 Gy in 2 Fr (SBRT) Control: 20 Gy in 5 Fr (3DRT)	**CPR**: 35% SBRT, 14% 3DRT *(p* = *0.0002)* **PPR**: 18% SBRT, 25% 3DRT	**CPR**: 32% SBRT, 16% 3DRT *(p* = *0.0036)* **PPR**: 9% SBRT, 16% 3DRT	One CTCAE grade 4 (vertebral compression fracture, 3DRT arm) No RIM
Ryu et al., 2023 [24]	Phase III	339	Exp.: 16 or 18 Gy in 1 Fr (SBRT) Control: 8 Gy in 1 Fr (3DRT)	**PPR**: 41.3% SBRT, 60.5% 3DRT *(p* = *0.99)*	**1-year PPR**: 57.6% SBRT, 55.3% 3DRT *(p* = *0.49)*	No RIM
Mercier et al., 2023 (abstract) [32]	Phase III	126	Exp.: 20 Gy in 1 Fr (SBRT) Control: 8 Gy in 1 Fr (3DRT)	**CPR**: 54% SBRT, 31% 3DRT *(p* = *0.048)*	/	No RIM Grade 2–3 AE: 15% SBRT *vs.* 14% 3DRT

*SBRT* stereotactic body radiotherapy, *exp.* experimental arm, *SBRT* stereotactic body radiotherapy, *3DRT* three-dimensional radiation therapy, *Fr* fraction, *RIM* radiation-induced myelopathy, *CTCAE* Common Terminology Criteria for Adverse Events, *CPR* complete pain response, *PPR* partial pain response, *AE* adverse events

#### Bone consolidation effect

Unlike other MDT, surgery and interventional radiology (IR) techniques (cementoplasty ± osteosynthesis) do not directly influence bone consolidation (defined as re-ossification promoted by an osteoblastic activation induced by treatment) but provide immediate stability, helping patients stand sooner and reducing decubitus complications [6]. In contrast, RT promotes bone recalcification, which enhances stabilization and LC, but this effect is delayed, typically manifesting 2 to 3 months post-treatment. A recent phase II randomized trial demonstrated that prophylactic RT significantly reduces SRE and hospitalizations in asymptomatic high-risk BM [33]. In this study, at 1 year, SRE occurred in 1.6% of patients receiving RT compared to 29% of those not receiving RT, with fewer hospitalizations for SREs in the RT group; high-risk factors for SRE include bulky lesion (size ≥ 2 cm), involvement of the hip, shoulder, or sacro-iliac region, one-third to two-thirds cortical damage of long bones, and junctional spinal disease or posterior arch involvement [33]. If no immediate stabilization procedure (surgery or IR) is performed, a prescription of an orthosis is recommended to provide structural support and stability to affected bones, reducing the risk of pathological fractures and improving the quality of life for patients by reducing the pain [34].

### Impact on local control (LC) and progression-free-survival (PFS)

The role of surgery in managing oligometastatic BM varies according to histological type. For solitary bone metastatic renal cell carcinoma (mRCC), complete metastasectomy and stabilization have been associated with improved survival and reduced local disease progression than standard care [35, 36]. In other histological types such as breast cancer, the place of surgery for oligometastatic BM is not consensual [37].

Recent studies suggest that thermal ablation methods, such as CA or RFA, can provide safe and effective LC of oligometastatic BM in selected patients. CA has a reported 1-year LC rate of 50–87%, with better outcomes for lesions < 2 cm and away from critical neurological structures [38].

SBRT has the strongest evidence for efficacy. For spinal lesions, SBRT achieves LC rates (evaluated by the criteria defined by the SPINO group) of approximately 80–90% at 1–2 years [26]. Non-spinal bone SBRT has even higher LC rates, with 94.6% at 1 year and 88% at 2 years according to recent meta-analyses [39, 40]. In *de novo* oligometastatic prostate cancer (PC), a prospective phase II trial of SBRT for up to 5 lesions found a biochemical response in 75% of patients [41]. For oligorecurrent PC, SBRT has demonstrated an improvement in progression-free survival (PFS) in 2 phase II studies [42, 43] and has delayed the need for androgen-deprivation therapy (ADT), potentially reducing adverse metabolic and cardiovascular effects [44]. In non-small-cell lung cancer (NSCLC), 3 phase II trials reported a three- to four-fold increase in PFS and overall survival (OS) with SBRT for oligometastases as a consolidative treatment [45–47]. The optimal timing for bone SBRT in oligometastatic NSCLC remains debated, but consolidative SBRT after initial systemic treatment is preferred to better identify responsive patients [48]. A recent phase II study found a benefit in terms of OS for 3DRT to asymptomatic BM after a median follow-up of 2.5 years, with a hazard ratio of 0.49 (95% confidence interval (CI) 0.27–0.89) [33], confirming the positive impact of early palliative care on OS in polymetastatic patients [49]. However, identifying oligometastatic patients who are likely to benefit from ablative MDT is challenging. ESTRO has proposed criteria for selecting suitable candidates for SBRT, applicable to other ablative techniques: number of metastases, histological type of the primary lesion, tumor size, tumor evolution profile, type of systemic treatment, and performance status [50]. Despite the promising role of all bone MDT for an ablative purpose, none has yet shown any benefit in terms of OS.

## MDT

### Surgery

#### Spinal metastases: neurosurgery

The neurosurgical management of spinal metastases involves evaluating neurological risk, mechanical instability, the number of metastases, the patient’s overall condition and his expected survival [11]. Several prognostic scores are available to guide physician to evaluated the expected survival [51, 52]. The objectives of surgery for spinal metastases can be categorized mainly into two groups: fixation to improve instability (fractures, pain) and decompression (with or without tumor resection) to relieve spinal cord compression. Typically, surgery is performed either individually for these objectives or as a combination of both. On rare occasions, complete tumor resection may be performed for renal cancer or thyroid cancer [53]. For MESCC, the standard treatment combines surgery with postoperative fractionated RT [54]. Minimally invasive spinal surgery (MISS) is a less complex and invasive alternative to traditional spinal surgery [10]. It focuses on stabilizing the spine and separating the spinal cord from tumors with minimal tissue removal, reducing morbidity. MISS is associated with reduced operative duration, shorter hospital stay, fewer complications, and expedited neurological recovery, along with a quicker initiation of adjuvant therapies [55]. A recent meta-analysis by the International Stereotactic Radiosurgery Society found that postoperative spine SBRT achieves a 1-year LC rate of 90% [56]. This strategy may be advantageous for patients with oligometastatic disease, previous loco-regional radiation, radioresistant histology, limited spinal involvement (≤ 2 segments), and an American Spinal Injury Association (ASIA) impairment scale score > A [57].

#### Non-spinal metastases: orthopedic surgery

For patients with a prolonged expected survival, arthroplasty should be considered due to its superior long-term functional outcomes [58]. For patients with a shorter expected survival, a simpler approach involving surgical osteosynthesis may be recommended [59]. The choice of the surgical procedure needs also consideration of the location of the bone tumor and the extent of the bone destruction (for example, arthroplasty preferred in case of peri-articular involvement). A complete radiographic evaluation of the affected bone is essential to evaluate the bone integrity before making any surgical decisions. Healthy bone is necessary to ensure the proper fixation and durability of the surgical hardware over time [60].

#### Complications of surgery

Oncologic surgery is associated with a high morbidity, including hemorrhage, surgical site infections, thromboembolic complications, and the risk of paraplegia [61]. These complications and their consequences (anemia, wound-healing disorders, and decubitus complication) could delay the initiation of systemic oncologic treatments, potentially compromising patient survival [62]. An interval of time should be observed between surgery and systemic treatment (as chemotherapy and anti-VEGF therapy) to limit the risk of post-operative complications [63].

Preoperative embolization, which involves endovascular occlusion of the primary arteries supplying a target lesion, can help prevent bleeding complications [64]. Its primary indication is prior to surgery for high vascularized metastases (such as kidney, melanoma, and thyroid cancer) to reduce intraoperative bleeding; this is ideally performed the day before or on the day of the surgery [65].

### Interventional radiology

#### Bone stabilization techniques

##### Principles of percutaneous cementoplasty

Cementoplasty is a minimally invasive procedure used for pain palliation and bone consolidation to prevent and manage SRE associated with osteolytic BM [66]. It involves the percutaneous injection of bone cement (polymethylmethacrylate) into the bone lesion. Cementoplasty is particularly appropriated for bones subjected to compression forces [67]. It can be applied to all vertebral levels (from C0 to the coccyx) and most extra-spinal BM treated with cementoplasty are in the pelvis [68]. While acetabular cementoplasty is highly effective, it is ideally performed only in the absence of fractures that extensively communicate with the coxo-femoral joint or involve significant damage to the acetabular fossa [68].

##### Association of cementoplasty with percutaneous osteosynthesis

**Fig. 1:**
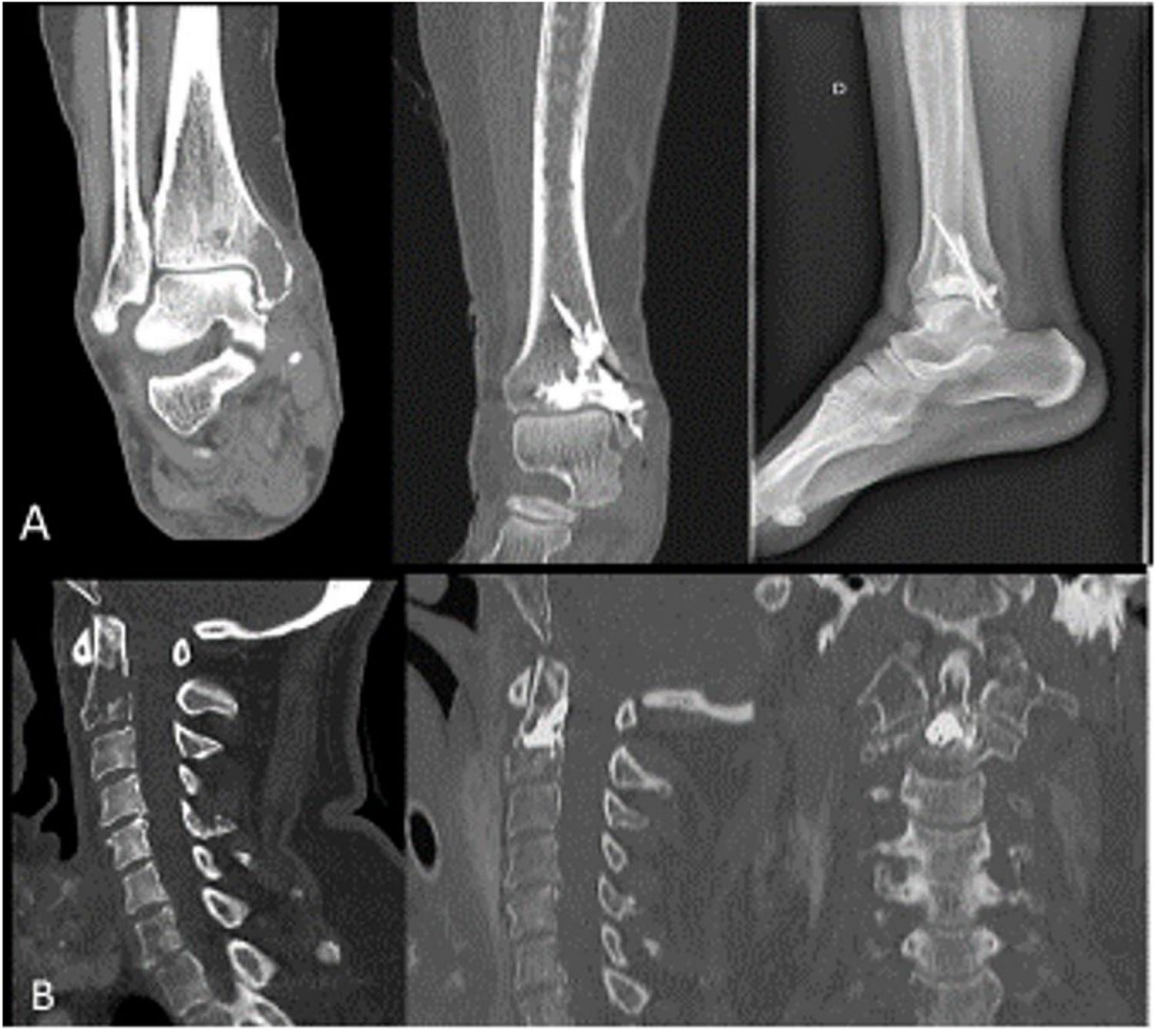
Examples of multimodal metastasis-directed therapy: **A** osteolytic lesion of the lateral malleolus of the right tibia with soft tissue involvement treated with radiotherapy, cementoplasty, and percutaneous osteosynthesis (left to right: coronal computed tomography (CT) scan of the lesion before any treatment; coronal CT scan of the right tibia after cementoplasty and osteosynthesis; sagittal radiography of the right ankle after treatment); **B** unstable osteolytic lesion of C2 treated with cementoplasty, screw fixation and radiotherapy (left to right: sagittal CT scan of the cervical spine with untreated lesion of C2; sagittal view after treatment; coronal view after treatment)

In case of extensive osteolytic destruction or lesions located in bones subjected to forces other than compression, cementoplasty is commonly associated with percutaneous osteosynthesis using screw fixation [69]. This strategy is particularly interesting for extensive iliac lesions or peripheral weight-bearing bones (femur, humerus, and tibia for example) [66, 70] and has favorable outcomes for femoral neck lesions [66, 71]. As an emerging combination, cementoplasty with screw fixation is generally reserved for patients with limited life expectancy, unfit for surgery. It can also be used in anatomically challenging areas, such as the pelvis or C2 [72]. Examples of this combined treatment is illustrated in Fig. 1.

#### Thermoablative (TA) procedures

### Radiofrequency ablation (RFA)

Needle-electrodes are introduced into the tumor and a current is applied which induces destruction via heat. Compared to CA, disadvantages include non-visualization of the ablation zone on CT-scan, intraprocedural pain and increased pain during the post-ablation period [73]. For this reason, RFA needs to be performed under general anesthesia. RFA is also associated with an increased risk of bone instability and therefore frequently associated with a cementoplasty. This combination provides sustained pain reduction [74]. Radiofrequency does not usually penetrate the cortex of healthy bone, which is an advantage within the vertebral body and avoids a risk to the spinal cord, contrary to CA. RFA also has the advantage of being able to cement the lesion immediately after treatment, whereas CA requires several days [74]. However, RFA can only be used on osteoclastic lesions due to the higher impedance of osteoblastic tumors rendering RFA ineffective [68].

#### Cryoablation (CA)

CA is a percutaneous thermal procedure that use extreme cold to destroy osteoblastic and osteoclastic lesions. The lesion is cooled by probes filled with argon, which induces cellular damage through the formation of intracellular ice crystals (according to the Joule-Thompson effect) [75]. Unlike other TA procedures, the extent of the “ice ball” can be monitored directly using CT scan or ultrasound to achieve better local control [76].

#### Electrochemotherapy (ECT)

ECT is a procedure combining reversible electroporation with local and/or intravenous administration of chemotherapy (bleomycin). Electroporation is a minimally invasive procedure in which high-intensity electric pulses are applied causing cell membrane damage and increasing tumor cell permeability to chemotherapy, thereby leading to mitotic cell death [77]. ECT seems to be a promising technique for recurrent MESCC following RT, with reported pain and neurological improvement in > 50% of cases [20].

#### Toxicities of interventional radiology techniques

Cement leakage is the most feared complication of cementoplasty. However, symptomatic leakage is rare (< 2%) but can result in MESCC, nerve root compression, or pulmonary cement embolism [12]. In the acetabulum, leakage of cement into the hip joint may result in significant functional impairment [68]. Transient post-procedural pain can occur in some cases. Careful management of the hemorrhagic risk is essential for patients undergoing anti-coagulant therapy, and preoperative embolization could also be considered. Extensive involvement of the posterior wall of the vertebral body is not an absolute contraindication, but cementoplasty should be performed by an experienced team and systematically combined with RT. Nerve or spinal cord proximity is not strictly a contra-indication of TA techniques, as innovative techniques have been developed to remove these vulnerable structures: hydro- or gas dissection, balloon interposition [66]. Absolute contraindications are notably severe coagulation disorders, general infection, infection at the puncture site, and neurological compression. The main complications associated with ECT are (grade < 3) transient acute radicular pain (25%) and prolonged radicular hypoesthesia (10%), followed by grade 3 definitive paraplegia immediately after the ECT or within the 2 weeks after the procedure; secondary fracture and grade 4 skin necrosis were also been reported [78]. ECT and TA procedures generally require a general anesthesia, with all the attendant risks.

## Radiotherapy

### Principles and doses

3DRT or IMRT is commonly used to treat painful BM. There is no significant difference in pain RR, acute toxicities, or risk of pathological fracture (< 5%) between patients receiving a single fraction of 8 Gy and those undergoing multiple fraction treatments (20 Grays (Gy)/5 Fr or 30 Gy/10 Fr) [21]. However, meta-analysis showed a significantly higher rate of re-irradiation in the single-fraction group (20% *vs*. 8%, *p* < 0.001) due to a longer LC and also a longer duration of pain relief in the multiple-fraction group [21].

For unstable BM, cauda equina syndrome, or SCC, a neurological evaluation is mandatory before considering RT. If the SINS exceeds 7, a stabilizing intervention should be considered before RT to reduce the risk of secondary fracture. Recent ESTRO guidelines recommend high-dose SBRT regimens, including schedules such as such as 1 × 20 Gy, 1 × 24 Gy, 2 × 12 Gy, 3 × 10 Gy, and 5 × 7 Gy [26]. Pre-SBRT MRI is strongly recommended to precisely delineate the spinal cord. In cases of MESCC, SBRT could be considered in association with neurosurgery. For non-spine BM, although the literature is less abundant, a similar treatment strategy may be applicable, with a preference for palliative SBRT in polymetastatic patients with a prolonged expected survival [23, 29]. Singh et al. reported a combined partial and complete pain RR of 87.7% at 3-month following SBRT [40].

### Combination with other modalities

Post-operative RT should be considered to reduce the risk of tumor dissemination and promote bone consolidation [79]. It stabilizes the surgical or IR hardware (prostheses and internal fixation devices) by inhibiting tumor regrowth and subsequent bone loss and reduces the frequency of local recurrence and post-intervention pain especially when surgery or IR procedure is incomplete [80]. Ideally, post-operative RT should be administered within 1 month following surgery, after satisfactory wound-healing, and should include the entire area of the surgical hardware [81].

When combining RT with percutaneous cementoplasty, RT might be preferentially administered before the cementoplasty, to minimize artifacts induced by cement during RT planning (especially in case of SBRT), but it is possible to administer this after cementoplasty. Additionally, RT facilitates tumor necrosis and improves the filling of the tumor by cement. Combining TA techniques with RT may enhance both the duration of pain relief and LC [17, 82].

Performing surgery or IR before RT provides an opportunity to obtain tumor tissue for further analysis.

### Toxicities

The frequency of radiation-induced myelopathy following spine SBRT is low (< 2%) [83]. Recent modeling analyses have reported the maximum point dose to the spinal cord, based on the number of fractions, to maintain the risk under 5% [84]. Vertebral compression fracture (VCF) is the most relevant toxicity after SBRT, the rate of which is reported to range from 10 to 20% [85]. The risk of VCF is influenced by the prescribed dose and the number of fractions, but rarely requires invasive management [85, 86]. Fractionated SBRT has not been associated with a higher risk of VCF compared to conventional RT, while single-fraction SBRT doses > 20 Gy appeared to be associated with an greater risk [24, 28]. Acute pain flare is common after SBRT, up to 30%, similar to 3DRT [87]. For non-spine bone SBRT, the main complication is rib (2.5%) and femur (1.9%) fracture [39]. Particular attention is needed when conventional RT or SBRT is performed in conjunction with systemic treatment, due to their potential radiosensitizing effect [63].

### Bone response assessment after MDT

Evaluation of treatment response after MDT is challenging. The Response Evaluation Criteria in Solid Tumor (RECIST) criteria is not adapted for BM. Post-surgical evaluation, including after procedures such as cementoplasty or percutaneous osteosynthesis, is often difficult or unfeasible due to artifacts caused by surgical equipment. RECIST assessment is also challenging following TA techniques. For instance, the RFA zone may not be visible on CT scans, and analysis can be hampered by the presence of cement; for CA, although the extent of the ablation zone is clearly visible on the CT scan, pseudoprogression due to induced necrosis can mislead morphological evaluation. After TA techniques, MRI may be preferred for assessing response by evaluating contrast enhancement of the lesion, or PET-CT may be used for metabolic evaluation.

Post-SBRT, evaluating morphological response is complicated by the occurrence of pseudo-progression several months after treatment. The SPIne response assessment in Neuro-Oncology (SPINO) group has published recommendations for response evaluation after spinal SBRT, suggesting that MRI is preferable over CT for assessing spinal tumor response [88]. Biopsy may be considered for persistent uncertainty. The University of Texas MD Anderson Cancer Center (MDACC) has developed its own criteria for BM, which are more suitable than RECIST for evaluating therapeutic response [89].

^18^F-FDG PET-CT has demonstrated strong performance for the evaluation of early therapeutic response for BM [5]. For instance, in breast cancer, Positron Emission Tomography Response Criteria In Solid Tumors (PERCIST) have proven superior to RECIST for accurately evaluating bone response. In addition, metabolic response is more closely correlated with PFS and disease-specific survival than morphologic response [90].

### Bone-targeted agents and oncological systemic treatment

Systemic approaches for the management of BM include targeted antitumor therapies and bone-targeted agents and anti-resorptive drugs being the cornerstone of management. Bisphosphonates and denosumab are bone resorption inhibitors and both prevent SRE [91, 92]. In PC, the benefit is observed exclusively in patients with metastatic castration-resistant PC (mCRPC), in whom there is a reported 36% reduction in the risk of SRE (*p* = 0.002) [93]. Several phase III studies have demonstrated the superiority of denosumab over zoledronic acid in reducing the frequency of SRE in bone metastatic breast and prostate cancers [94, 95]. However, the difference has not been observed in other cancer types [96, 97]. Bone resorption inhibitors have also shown a benefit in terms of SRE and OS for in bone metastatic lung cancer and myeloma [98, 99].

Sometimes, systemic therapies are effective on bone, positively impacting the prevention of SRE; for uncomplicated and mildly painful BM, in some particular cases, omission of MDT may be considered. A “wait-and-see” strategy should always be supported by the prescription of antiresorptive treatment, and patients should be closely monitored with early bone reassessment 6 weeks after the initiation of systemic treatment or if pain or neurological symptoms arise. For example, abiraterone acetate and enzalutamide in mCRPC have been shown to improve SRE prevention (HR 0.61, 95% CI [0.48–0.79] and 0.71, 95% CI [0.63–0.80], respectively) [100]. Tyrosine kinase inhibitors (TKI) may also modulate osteoblast activity, as reported for EGFR mutated adenocarcinoma lung cancers. For instance, in the COMET- 1 and METEOR trials, cabozantinib was associated with a reduction in SRE and an increased median time to the first SRE [101, 102].

Conversely, chemotherapy and hormonotherapy are more likely to cause bone damage through both direct and indirect mechanisms. The most studied indirect effect is the loss of ovarian function in females, which can lead to rapid bone loss [103]. Myelosuppression, induced by chemotherapy but also by the corticosteroids often used in metastatic disease, contributes to bone demineralization through increased bone resorption and decreased osteoblast activity [104].

## Discussion

**Fig. 2:**
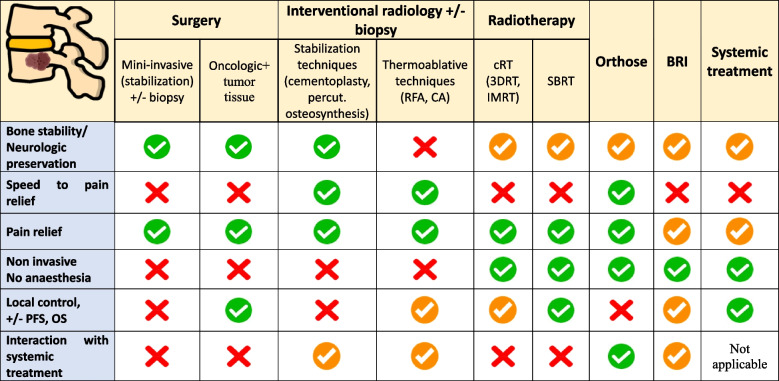
Summary of the main advantages and disadvantages of metastasis-directed therapies (MDT) in bone metastases management. This table describes the suitability of a technique for a given clinical objective. A green checkmark means that the technique makes it possible to achieve the objective. A orange checkmark means that the objective is partially met with the technique. The red cross means that the technique is unable to achieve the objective. OS: overall survival; PFS: progression-free survival; cRT: conventional radiotherapy; 3DRT: tridimensional radiotherapy; IMRT: intensity modulated radiation therapy; CRA: cryotherapy ablation; RFA: radiofrequency ablation; SBRT: stereotactic body radiation therapy; BRI: bone resorption inhibitor

**Fig. 3:**
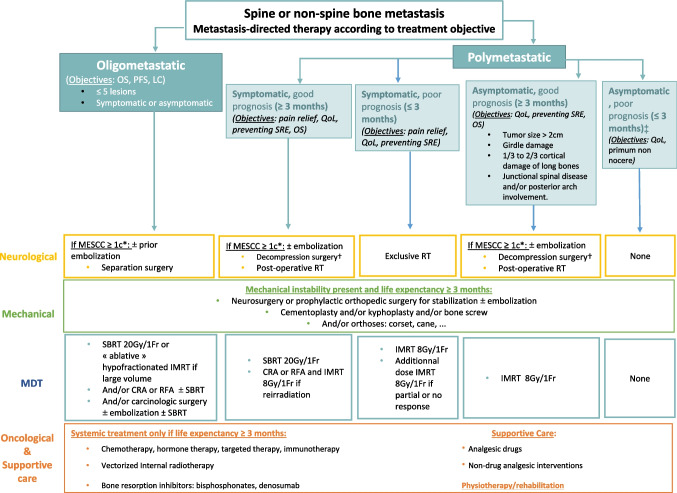
MDT-NOMS algorithm for metastasis-directed therapy on bone according to treatment objective. OS: overall survival; PFS: progression-free survival; LC: local control; SRE: skeletal-related events; IMRT: intensity modulated radiation therapy; CRA: cryotherapy ablation; RFA: radiofrequency ablation; SBRT: stereotactic body radiotherapy; QoL: quality of life; MESCC: metastatic epidural spinal cord compression; RT: radiotherapy; Gy: grays. ^*^Bilsky classification. ^†^Separation surgery + SBRT may be discussed in selected cases of patients with radioresistant primary tumor. ^‡^In selected cases, a mini-invasive procedure (surgery or interventional radiology) may be discussed in case of symptomatic instability profoundly impacting quality of life

BM, due to the frequency of SRE and their associated morbidity and mortality, require a multidisciplinary approach for management. MDT, systemic treatment, anti-resorptive therapies, and supportive care can be proposed to prevent or treat SRE in alignment with the overall oncological strategy. The choice of a specific treatment depends on several factors: clinical situation (invasive procedure, interaction with systemic treatment), presence of functional risks (instability or neurological issues), patient characteristics (symptomatic or asymptomaic BM, life expectancy, Karnofsky Performance Score), and treatment objectives (pain relief, local control; Fig. 2). Based on some decision points of the previous neurologic, oncologic, mechanical instability, and systemic disease (NOMS) framework [4], we propose an updated version (MDT-NOMS) taking recent innovations into account (Fig. 3). These modalities complement each other; however, careful assessment is crucial to provide optimal treatment, and the bone metastatic multidisciplinary tumor board (BM^2^TB) plays an important role for promoting an integrated approach to manage BM with multimodal treatments [60].

Certain limitations in the multimodal management of BM need to be discussed. For instance, while nearly all university hospitals or comprehensive cancer centers have developed a dedicated BM^2^TB, access to such boards may be limited in smaller facilities. Moreover, these centers may lack technical resources or specialized expertise, which could result in simplified management. Local physicians should have the option to consult a BM^2^TB at a regional reference center for the presentation or management of these patients. In addition, the delay in accessing MDT could also restrict treatment options. Furthermore, the level of evidence for MDT in the literature is generally weak, except for RT, and outcomes for evaluating pain relief or LC are heterogeneous, complicating comparisons.

## Conclusion

The therapeutic arsenal available for the management of spinal and extra-spinal BM is varied. It includes both local treatments (RT, interventional radiology, orthoses, and surgery) and systemic therapies (oncological treatments, bone-targeted agents, and supportive care). The goal is to optimize local control to prevent SRE, while limiting treatment-related morbidity. In this respect, SBRT plays a key role, offering excellent local control and rapid, lasting pain relief. The management of BM proposed in this article may help clinicians to identify the best therapeutic sequence, according to the patient’s oncological status and expected clinical benefits. It is by considering the stability of the lesion, the degree of extension of the disease, in conjunction with the patient’s co-morbidities, that the optimal combination of irradiation modality, interventional radiology, and surgery can be correctly determined. These therapeutic modalities are most often complementary rather than competitive in the management of bone metastases. We need more than ever therapeutic trials of strategies combining the different modalities together in order to better evaluate them.

## Data Availability

No datasets were generated or analysed during the current study.

## Abbreviations

ASTRO: American Society for Therapeutic Radiology and Oncology
BM: Bone metastases
BM^2^TB: Bone Metastatic Multidisciplinary Tumor Board
CA: Cryoablation
CT: Computed tomography
ESTRO: European Society for Therapeutic Radiology and Oncology
FDG: Fluorodeoxyglucose
GEMO: European Study Group of Bone Metastases
Gy: Grays
LC: Local control
mCRPC: Metastatic castration-resistant prostate cancer
MDT: Metastasis-directed therapy
MESCC: Metastatic epidural spinal cord compression
MISS: Minimally invasive spinal surgery
MRI: Magnetic resonance imaging
NOMS: Neurologic, oncologic, mechanical instability, and systemic disease
OS: Overall survival
PC: Prostate cancer
PERCIST: Positron Emission Tomography Response Criteria In Solid Tumors
PET: Positron emission tomography
PFS: Progression-free survival
PS: Performance status
QoL: Quality of life
RECIST: Response Evaluation Criteria in Solid Tumor
RFA: Radiofrequency ablation
RR: Response rate
RT: Radiotherapy
SBRT: Stereotactic body radiotherapy
SINS: Spinal Instability Neoplastic Score
SRE: Skeletal-related events
3DRT: Three-dimensional radiation therapy
